# Pulmonary diffusing capacity for carbon monoxide and nitric oxide after COVID‐19: A prospective cohort study (the SECURe study)

**DOI:** 10.1113/EP091757

**Published:** 2024-03-26

**Authors:** Anna Agnes Lytzen, Thora Wesenberg Helt, Jan Christensen, Thomas Kromann Lund, Anna Kalhauge, Frederikke Falkencrone Rönsholt, Daria Podlekavera, Elisabeth Arndal, Anne‐Mette Lebech, Birgitte Hanel, Terese L. Katzenstein, Ronan M. G. Berg, Jann Mortensen

**Affiliations:** ^1^ Centre for Physical Activity Research Copenhagen University Hospital—Rigshospitalet Copenhagen Denmark; ^2^ Department of Clinical Physiology and Nuclear Medicine Copenhagen University Hospital—Rigshospitalet Copenhagen Denmark; ^3^ Department of Occupational Therapy and Physiotherapy Copenhagen University Hospital—Rigshospitalet Copenhagen Denmark; ^4^ Department of Cardiology, Section for Lung Transplantation Copenhagen University Hospital—Rigshospitalet Copenhagen Denmark; ^5^ Department of Radiology Copenhagen University Hospital—Rigshospitalet Copenhagen Denmark; ^6^ Department of Infectious Diseases Copenhagen University Hospital—Rigshospitalet Copenhagen Denmark; ^7^ Department of Respiratory Medicine and Infectious Diseases Copenhagen University Hospital—Bispebjerg Hospital Copenhagen Denmark; ^8^ Department of Otorhinolaryngology Copenhagen University Hospital—Rigshospitalet Copenhagen Denmark; ^9^ Department of Biomedical Sciences, Faculty of Health and Medical Sciences University of Copenhagen Copenhagen Denmark; ^10^ Neurovascular Research Laboratory, Faculty of Life Sciences and Education University of South Wales Pontypridd UK; ^11^ Department of Medicine The National Hospital Torshavn Faroe Islands; ^12^ Department of Clinical Medicine, Faculty of Health and Medical Sciences University of Copenhagen Copenhagen Denmark

**Keywords:** diffusion, long COVID, SARS‐CoV‐2

## Abstract

Many patients exhibit persistently reduced pulmonary diffusing capacity after coronavirus disease 2019 (COVID‐19). In this study, dual test gas diffusing capacity for carbon monoxide and nitric oxide (*D*_L,CO,NO_) metrics and their relationship to disease severity and physical performance were examined in patients who previously had COVID‐19. An initial cohort of 148 patients diagnosed with COVID‐19 of all severities between March 2020 and March 2021 had a *D*_L,CO,NO_ measurement performed using the single‐breath method at 5.7 months follow‐up. All patients with at least one abnormal *D*_L,CO,NO_ metric (*n* = 87) were revaluated at 12.5 months follow‐up. The *D*_L,CO,NO_ was used to provide the pulmonary diffusing capacity for nitric oxide (*D*_L,NO_), the pulmonary diffusing capacity for carbon monoxide (*D*_L,CO,5s_), the alveolar–capillary membrane diffusing capacity and the pulmonary capillary blood volume. At both 5.7 and 12.5 months, physical performance was assessed using a 30 s sit‐to‐stand test and the 6 min walk test. Approximately 60% of patients exhibited a severity‐dependent decline in at least one *D*_L,CO,NO_ metric at 5.7 months follow‐up. At 12.5 months, both *D*_L,NO_ and *D*_L,CO,5s_ had returned towards normal but still remained abnormal in two‐thirds of the patients. Concurrently, improvements in physical performance were observed, but with no apparent relationship to any *D*_L,CO,NO_ metric. The severity‐dependent decline in *D*_L,NO_ and *D*_L,CO_ observed at 5.7 months after COVID‐19 appears to be reduced consistently at 12.5 months follow‐up in the majority of patients, despite marked improvements in physical performance.

## Abstract (highlights)

- **What is the central question of this study?**
  To what extent do changes in pulmonary diffusing capacity observed 6 months after coronavirus disease 2019 (COVID‐19) persist after a year?
- **What is the main finding and its importance?**
  In patients who have had COVID‐19, the changes in pulmonary diffusing capacity for carbon monoxide and nitric oxide, the alveolar–capillary membrane diffusing capacity and pulmonary capillary blood volume observed at approximately 6 months follow‐up largely persist 1 year after the acute disease, despite a marked improvement in physical performance.

## 1 INTRODUCTION

After the initial surge of the coronavirus disease 2019 (COVID‐19) pandemic, it became evident that the pulmonary effects of the disease often lasted far beyond the immediate severe acute respiratory syndrome coronavirus 2 (SARS‐CoV‐2) infection (Crook et al., 2021). Besides common pulmonary symptoms, such as breathlessness, chest pain and fatigue, grouped under the term ‘long COVID’, numerous studies have reported a severity‐dependent reduction in pulmonary diffusing capacity (*D*_L_) (Huntley et al., 2022; Katzenstein et al., 2022; Watanabe et al., 2022).

Morphologically, the post‐COVID reduction in *D*_L_ has been shown to be caused by a fibrosis‐like restrictive lung disease rather than pulmonary vascular disease (Katzenstein et al., 2022). These changes appear to be reversible, at least in part, although residual parenchymal damage is often observed in severe cases (Lee et al., 2022; Torres‐Castro et al., 2021; Zhao et al., 2020). Functionally, dual test gas diffusing capacity assessments using carbon monoxide (CO) and nitric oxide (NO) (i.e., *D*_L,CO,NO_), performed at 2 to 4 months follow‐up, suggest that the impairment of pulmonary diffusion is caused mainly by alveolar–capillary membrane diffusing capacity (*D*_M_) changes following mild‐to‐moderate COVID‐19 (Barisione & Brusasco, 2021). In contrast, a combination of *D*_M_ and capillary blood volume (*V*_C_) changes appear to be involved following severe COVID‐19 (Núñez‐Fernández et al., 2021; Seccombe et al., 2023). At present, it is unknown whether the recovery of diffusing capacity is caused by an increase in *D*_M_ and/or *V*_C_ and whether such changes are related to changes in physical performance. Furthermore, it is unknown whether the post‐COVID changes in diffusing capacity are associated with an impairment of alveolar–capillary reserve; that is, the ability to recruit and distend pulmonary capillaries (Behnia et al., 2017; Hsia et al., 1994).

The present paper is based on data from the Danish SECURe (Sequelae of COVID‐19 at Copenhagen University Hospital—Rigshospitalet) study, a prospective cohort study designed to track post‐COVID complications using comprehensive clinical, physiological and chest imaging evaluations in both hospitalized and non‐hospitalized COVID‐19 patients (Katzenstein et al., 2022). Here, we explored: (1) the degree to which the *D*_L,CO,NO_ metrics were reduced at 5.7 months follow‐up in the SECURe cohort; (2) the extent to which the post‐COVID decline in *D*_L,CO,NO_ persisted up to 12.5 months follow‐up, including the extent to which any such changes were caused by *D*_M_ and *V*_C_, respectively; and (3) whether any such changes were related to changes in physical performance.

## 2 MATERIALS AND METHODS

### 2.1 Ethical approval

The study conformed to the standards set by the most recent version of the *Declaration of Helsinki* (except for registration in a database) and was approved by the Danish Data Protection Agency and the Regional Ethical Committee of the Capital Region of Denmark (file no. H‐20028792). All study participants provided oral and written informed consent.

### 2.2 Study design and setting

The SECURe study is a prospective cohort study of individuals who were diagnosed with SARS‐CoV‐2 confirmed by PCR testing by an oral or nasopharyngeal swab. The study was conducted as a collaboration between several departments at University Hospital Copenhagen—Rigshospitalet, a specialized University Hospital in Denmark serving mainly as a tertiary health‐care service.

### 2.3 Recruitment

All consecutive COVID‐19 patients admitted to the Department of Infectious Diseases at University Hospital Copenhagen—Rigshospitalet from March 2020 to March 2021 were invited to participate. At discharge and/or during the first post‐discharge telephone consultation, patients were informed about the study. The telephone consultation was provided as a standard procedure to all COVID‐19 patients who had been admitted. SARS‐CoV‐2‐infected participants without need for hospitalization were also invited to participate. For admitted patients, the initial SECURe study visit was aimed at being scheduled 3–4 months post‐discharge, whereas for non‐admitted participants, it was scheduled for 3–4 months after testing positive for SARS‐CoV‐2 (Katzenstein et al., 2022). In the present paper, we report on all participants (*n* = 148) who had a *D*_L,CO,NO_ measurement performed at 5.7 months follow‐up. All patients who had at least one abnormal *D*_L,CO,NO_ metric (*n* = 87) were re‐evaluated at ∼12.5 months follow‐up.

### 2.4 Clinical scores

Participants were classified as group 1 (asymptomatic), group 2 (mild), group 3 (moderate), group 4 (severe) or group 5 (critical), according to the clinical features during the acute SARS‐CoV‐2 infection (Gandhi et al., 2020). To assess symptom severity during follow‐up, the chronic obstructive pulmonary disease (COPD) assessment test (CAT) was used, which is a questionnaire designed to assess the impact of COPD on everyday life activities and enables the evaluation of changes over time (Jones, Harding, Wiklund et al., 2009). The questionnaire focuses mainly on respiratory symptoms, such as shortness of breath, cough, chest pain and limitations in physical abilities; however, questions also relate to energy levels and sleep patterns (Jones, Harding, Berry et al., 2009). Comorbidity burden was assessed at baseline using the Charlson comorbidity index (Charlson et al., 1987).

### 2.5 Physical performance

Physical performance was assessed using 30 s sit‐to‐stand (STS) muscle strength test and 6 min walk test (6MWT) (Alcazar et al., 2018; Peeters & Mets, 1996). The STS test was conducted in accordance with standard test procedures. The 6MWT was performed on a 30 m track. Both STS and 6MWT were reported as a percentage of predicted based on the empirical equations provided elsewhere (Enright & Sherrill, 1998; Suetta et al., 2019).

### 2.6 Lung function testing

Lung function testing included dynamic spirometry, body plethysmography and single‐breath measurement of *D*_L,CO_ in accordance with European Respiratory Society (ERS) guidelines (Bhakta et al., 2023; Graham et al., 2019). The tests were conducted at the Department of Clinical Physiology and Nuclear Medicine at University Hospital Copenhagen—Rigshospitalet using an integrated system (MasterScreen; Vyaire Medical, Würzburg, Germany). Before measurements, standing height (to the nearest 1 mm), body mass (to the nearest 100 g) and haemoglobin (Hb) concentration (to the nearest 0.1 mM) were obtained. The [Hb] was measured in capillary blood using an enzymatic system (HemoCue, Hb 201+; Copenhagen, Denmark). The variables reported in the present study are forced expiratory volume in 1 s (FEV_1_), forced vital capacity (FVC), FEV_1_/FVC, total lung capacity (TLC), residual volume (RV) and [Hb]‐corrected *D*_L,CO_. The *D*_L,CO_ was based on an 8–12 s breath‐hold (denoted as *D*_L,CO,10s_) using a gas mixture of 0.3% CO, 0.3% CH_4_, 20.9% O_2_ and balance N_2_. Values were reported as a percentage of predicted, except for FEV_1_/FVC, which was reported as a raw value. Reference values were based on height, sex and age as appropriate (Stanojevic et al., 2022).

### 2.7 Dual test gas diffusing capacity for CO and NO

The *D*_L,CO,NO_ was assessed in the seated upright position, adhering to the ERS task force recommendations (Zavorsky et al., 2017). The procedure commenced with tidal breathing, transitioning to a maximal expiration to residual volume, followed by a swift maximal inhalation to total lung capacity. During this, subjects inhaled a gas mix mixture of 0.2% CO, 50 p.p.m. NO, 6.3% He, 20.9% O_2_ and balance N_2_. This was followed by a stable 4–8 s breath‐hold at total lung capacity, then a forceful, uninterrupted maximal expiration. The *D*_L,CO_ measurements obtained by this method are denoted as *D*_L,CO,5s_.

Manoeuvres were deemed acceptable if the inspired volume from TLC was ≥90% of FVC or the plethysmography‐derived vital capacity, or if it was ≥85% of the FVC or plethysmography‐based vital capacity with a *V*_A_ within either 200 mL or 5% of the highest alveolar volume (*V*_A_) from previous acceptable manoeuvres (Zavorsky et al., 2017). Repeatability was confirmed on the basis of two consistent readings that fell within 5.8 mmol min^−1^ kPa^−1^ (17.4 mL min^−1^ mmHg^−1^) for *D*_L,NO_, 1.0 mmol min^−1^ kPa^−1^ (2.98 mL min^−1^ mmHg^−1^) for *D*_L,CO,5s_, 11.2 mmol min^−1^ kPa^−1^ (33.5 mL min^−1^ mmHg^−1^) for *D*_M_ and 10 mL for *V*_C_ (Zavorsky et al., 2017). Readings were repeated post‐washout until repeatability was achieved and were limited to 12 attempts. The average of two manoeuvres that satisfied the repeatability criteria was reported for both *D*_L,NO_ and *D*_L,CO,5s_. When these criteria were not met, the data from the best manoeuvre were used, with deviations highlighted for judicious evaluation.

Participants with at least one abnormal *D*_L,CO,NO_ metric (*D*_L,CO,5s_, *D*_L,NO_, *D*_M_ or *V*_C_) at 12.5 months follow‐up were asked to perform an additional measurement in the supine position when practically possible (Madsen et al., 2023). For this, participants were placed in the supine position, with slight head elevation using a pillow for comfort; after 15 min of rest in this position, the *D*_L,CO,NO_ measurements were repeated using the same approach and criteria as described above.

### 2.8 Calculations

The values of *D*_M_ and *V*_C_ were calculated from *D*_L,CO,NO_ measurements using the following equations (Munkholm et al., 2018):

$$\;D_{M}=\;\frac{\frac{1}{\alpha }\;-\;\frac{1}{k}}{\frac{1}{D_{L,\mathrm{NO}}\;}-\;\frac{1}{k\times D_{L,\mathrm{CO},5s}}}$$

$$\;V_{C}=\;\frac{\left(\frac{1}{\theta_{\mathrm{CO}}}\right)\left(1-\frac{\alpha }{k}\right)}{\frac{1}{D_{L,\mathrm{CO},5s\;}}\;-\;\frac{\alpha }{D_{L,\mathrm{NO}}}}$$

where α is the *D*_M_ ratio (*D*_M,NO_/*D*_M,CO_), which is assumed to be 1.97, and *k* is the ratio between the specific conductance of the two test gases (θ_NO_/θ_CO_). Here, θ_NO_ was assumed to have a finite value of 1.51 mL blood min^−1^ kPa^−1^ (mmol NO)^−1^ [4.5 L blood min^−1^ mmHg^−1^ (mL NO)^−1^] (Carlsen & Comroe, 1958), and θ_CO_ was calculated as:

$\theta \mathrm{CO}=\frac{1}{3.88\;\mathrm{mmo}l_{\mathrm{CO}}\cdot \min\cdot \mathrm{kPa}\cdot m{L_{\mathrm{blood}}}^{-1}+0.092\;\mathrm{mmo}l_{\mathrm{CO}}\cdot \min\cdot m{L_{\mathrm{blood}}}^{-1}\cdot 13.33\;\mathrm{kPa}}\cdot \frac{\left[\mathrm{Hb}\right]}{9.06\mathrm{mM}}$where [Hb] is the concentration of haemoglobin in capillary blood (mM).

Values of *D*_L,NO_, *D*_L,CO,5s_, *D*_M_ and *V*_C_ were reported as raw values, as a percentage of predicted (%pred) and as *z*‐scores using established reference equations to normalize for height, sex and age (Munkholm et al., 2018).

### 2.9 Statistical analyses

All data were entered in REDCap (v.13.7.14 © 2023 Vanderbilt University, TN, USA). Statistical analyses were performed using STATA v.18 (StataCorp, Stata Statistical Software, College Station, TX, USA). Participant characteristics, CAT score, Charlson comorbidity index score, physical performance, lung function and *D*_L,CO,NO_ metrics (including the number of abnormal scores and change from abnormal to normal) were summarized as percentage (*n*), mean (SD) for normally distributed variables or median [interquartile range] for non‐normally distributed variables based on visual inspection of histograms and probability plots. Differences between severity groups were assessed using Fisher's exact test for sex, one‐way ANOVA for normally distributed data and the Kruskal–Wallis test for non‐normally distributed data. An abnormal diffusion score was defined as a *z*‐score < −1.65. For the normally distributed variables, differences over time within COVID‐19 severity groups were calculated using Student's paired *t*‐test. For the non‐normally distributed variables, bootstrap with bias correction was used to calculate 95% confidence intervals (Konietschke & Pauly, 2014; Tibbe & Montoya, 2022). Least squares linear regression was used to predict the physical performance (STS and 6MWT) from the change in *D*_L,NO_%pred and *D*_L,CO,5s_%pred, respectively, and correlation coefficients were calculated using Pearson's product–moment correlation coefficient. For all data, a two‐sided *P* < 0.05 was considered statistically significant.

## 3 RESULTS

### 3.1 Participant characteristics

Demographics and clinical characteristics according to disease severity at 5.7 months (median 170 days, interquartile range 46– 263 days) follow‐up are provided in Table 1. Of note, greater disease severity was associated with lower lung function metrics, including FEV_1_, FVC, TLC, RV and a lower *D*_L,CO,10s_.

**TABLE 1:** Participant demographics and clinical characteristics.

Parameter	*n*	All	Group 1 (*n* = 1)	Group 2 (*n* = 38)	Group 3 (*n* = 22)	Group 4 (*n* = 66)	Group 3 (*n* = 21)	Between‐group *P*‐value
Sex, *n* (% male)	148	79 (53)	0 (0)	12 (32)	7 (32)	45 (68)	15 (71)	<0.001
Age at diagnosis, years, *n* (SD)	148	53.8 (15.1)	43	44.9 (12.8)	46.8 (17.4)	59.9 (12.9)	58.4 (12.2)	<0.001
Body mass, kg	148	82.8 [71.3; 96.9]	71.6	73.2 [65.4; 81.2]	77.5 [67.6; 94.6]	88.6 [80.6; 101]	96.3 [74.7; 106]	<0.001
Height, cm	148	172.0 (10.7)	167.8	169.0 (9.7)	169.8 (11.5)	173.8 (10.5)	174.0 (11.3)	0.15
BMI, kg/m^2^	148	27.6 [24.4; 31.8]	25.4	25.6 [22.5; 28.6]	27.2 [22.9; 34.6]	29.4 [25.2; 33.0]	30.2 [26.2; 31.0]	0.010
CAT score	147	6 [2; 10]	10	6 [2; 10]	6 [2; 12]	5 [1; 9]	5 [4; 10]	0.59
CCI score	147	2 [1; >3]	1	1 [0; 2]	1 [0; 2]	3 [2; >3]	3 [2; >3]	<0.001
Lung function metrics								
FEV_1_%pred	148	108.4 [96.0; 121.2]	103.1	112.0 [101.0; 119.2]	109.6 [96.1; 121.7]	109.8 [93.4; 124.5]	97.9 [90.6; 119.2]	0.68
FVC%pred	148	114.2 [98.0; 124.4]	125.8	120.3 [109.6; 127.5]	114.4 [98.1; 124.0]	114.0 [95.6; 121.6]	103.1 [91.4; 119.5]	0.029
FEV_1_/FVC	148	80.0 [74.7; 83.1]	70.7	78.7 [73.5; 82.7]	81.3 [77.9; 84.1]	79.9 [75.6; 83.1]	80.6 [77.6; 83.6]	0.31
TLC%pred	147	101.8 [90.7; 110.3]	117.0	109.9 [101.4; 116.3]	104.9 [91.1; 110.3]	100.1 [90.8; 106.5]	86.9 [82.1; 101.8]	<0.001
RV%pred	147	88.6 [78.0; 102.1]	108.6	94.8 [79.6; 106.8]	87.4 [82.1; 99.5]	89.1 [79.6; 102.1]	70.5 [63.2; 81.7]	<0.001
D_L,CO,10s_%pred	148	79.5 [72.4; 89.2]	76.7	83.3 [77.6; 99.4]	85.1 [75.9; 94.0]	78.2 [71.6; 85.2]	66.1 [56.8; 76.4]	<0.001

*Note*: Assessments were made at 5.7 months follow‐up after COVID‐19. Values are presented as percentage (*n*), mean (SD) for normally distributed data or median [interquartile range] for non‐normally distributed data. Values of CCI higher than three were recorded as four for calculation of the median. Group 1, asymptomatic COVID‐19; group 2, mild COVID‐19; group 3, moderate COVID‐19; group 4, severe COVID‐19; group 5, critical COVID‐19. Abbreviations: CAT score, chronic obstructive pulmonary disease assessment test; CCI, Charlson comorbidity index; *D*_L,CO,10s_%pred, pulmonary diffusing capacity for carbon monoxide (during a 10 s breath‐hold) as a percentage of that predicted according to age, height and sex; FEV_1_%pred, forced expiratory volume in the first second as a percentage of that predicted according to age, height and sex; FEV_1_/FVC, ratio of forced expiratory volume in the first second to forced vital capacity; FVC_1_%pred, forced vital capacity as a percentage of that predicted according to age, height and sex; RV%pred, residual volume as a percentage of that predicted according to height and sex; TLC%pred, total lung capacity as a percentage of that predicted according to height and sex. Differences between severity groups were assessed using Fisher's exact test for sex, one‐way ANOVA for normally distributed data and the Kruskal–Wallis test for non‐normally distributed data.

### 3.2 Dual test gas diffusing capacity for CO and NO

Of the 148 patients initially included, 87 (59%) completed follow‐up *D*_L,CO,NO_ at 12.5 months (median 375 days, interquartile range 328–581 days), of whom only one patient was classified as group 1. At 5.7 months follow‐up, group 4 and 5 patients generally had a higher rate of abnormal *D*_L,NO_ and *D*_L,CO,5s_ and *V*_C_ scores than the other severity groups, and the rate of abnormal *D*_M_ was higher in group 5 than in the other groups (Table 2). A largely similar distribution of abnormal scores was observed in the 87 patients who completed 12.5 months follow‐up

**TABLE 2:** Number of abnormal pulmonary diffusing capacity scores.

		Number of abnormal scores [% (*n*)]					
Parameter	*n*	All groups	Group 1	Group 2	Group 3	Group 4	Group 5
*D*_L,NO_							
5.7 months	148	59 (87)	100 (1)	47 (18)	45 (10)	64 (42)	76 (16)
12.5 months	87	66 (57)	100 (1)	50 (7)	71 (10)	63 (26)	76 (13)
*D*_L,CO,5s_							
5.7 months	148	63 (93)	100 (1)	53 (20)	50 (11)	67 (44)	81 (17)
12.5 months	87	74 (64)	100 (1)	57 (8)	79 (11)	76 (31)	76 (13)
*D*_M_							
5.7 months	120	39 (47)	100 (1)	33 (9)	37 (7)	37 (20)	53 (10)
12.5 months	48	44 (21)	100 (1)	57 (4)	50 (4)	33 (8)	50 (4)
*V*_C_							
5.7 months	120	61 (73)	0 (0)	41 (11)	58 (11)	72 (39)	63 (12)
12.5 months	48	71 (34)	0 (0)	57 (4)	75 (6)	75 (18)	75 (6)

*Note*: Values are presented as % (*n*). Group 1, asymptomatic COVID‐19; group 2, mild COVID‐19; group 3, moderate COVID‐19; group 4, severe COVID‐19; group 5, critical COVID‐19. Abbreviations: *D*_L,CO,5s_, pulmonary diffusing capacity for carbon monoxide (during a 5 s breath‐hold); *D*_L,NO_, pulmonary diffusing capacity for nitric oxide; *D*_M_, alveolar–capillary membrane diffusing capacity; V_C_, pulmonary capillary blood volume.

The *D*_L,CO,NO_ metrics presented as a percentage of predicted are provided in Table 3, and changes from 5.7 to 12.5 months are shown in Table 4. The absolute values and *z*‐scores are provided in the Online Supplemental File. Overall, there was a small increase in both *D*_L,NO_%pred and *D*_L,CO,5s_%pred, of which the former did not show any distinct pattern with disease severity, while the latter was mostly observed in groups 4 and 5. However, no distinct change in either *D*_M_ or *V*_C_ was observed, either in the overall group of 87 patients or in any of the disease severity groups.

**TABLE 3:** Pulmonary diffusing capacity and physical performance at 5.7 and 12.5 months follow‐up.

		All groups		Group 1		Group 2		Group 3		Group 4		Group 5	
Parameter	*n*	5.7 months	12.5 months	5.7 months	12.5 months	5.7 months	12.5 months	5.7 months	12.5 months	5.7 months	12.5 months	5.7 months	12.5 months
*D*_L,NO_%pred	87	71.5 [63.8; 78.6]	76.0 [67.7; 83.6]	76.6	79.1	75.6 [69.9; 83.6]	80.6 [74.3; 85.8]	74.1 [62.3; 83.8]	76.4 [63.9; 82.2]	71.6 [66.5; 77.9]	75.2 [70.4; 84.6]	64.5 [61.3; 70.4]	67.7 [64.0; 81.4]
*D*_LCO,5s_%pred	87	70.5 [64.6; 77.9]	73.6 [66.2; 80.6]	77.9	77.4	76.2 [70.5; 79.9]	80.1 [73.7; 82.5]	73.5 [64.3; 80.3]	72.6 [66.2; 81.6]	70.0 [65.8; 76.7]	73.6 [68.1; 79.8]	65.1 [55.3; 71.2]	65.1 [59.3; 74.4]
*V*_C_%pred	41	67.3 (16.1)	69.6 (15.1)	79.6	81.8	78.0 (11.4)	78.8 (7.5)	70.3 (10.5)	70.7 (9.8)	63.9 (18.1)	67.2 (18.5)	62.9 (16.3)	65.9 (12.1)
*D*_M_%pred	41	75.5 (18.4)	77.4 (19.5)	74.4	77.0	75.1 (17.9)	76.2 (15.5)	83.1 (20.6)	78.5 (22.0)	76.0 (19.3)	78.9 (22.7)	67.0 (15.3)	73.3 (13.5)
STS%pred	36	63.0 [53.8; 68.7]	75.3 [62.5; 86.6]	53.2	74.5	68.0 [63.2; 68.2]	77.3 [68.5; 77.7]	61.7 [54.4; 66.5]	68.0 [56.8; 91.8]	63.0 [51.7; 73.1]	70.8 [61.4; 96.1]	61.7 [46.7; 76.1]	81.5 [71.4; 87.6]
6MWT%pred	37	95.0 [81.3; 107.7]	101.9 [92.8; 120.6]	90.9	95.6	102.4 [89.0; 107.1]	100.4 [88.5; 125.0]	82.8 [74.5; 91.2]	89.4 [85.9; 92.8]	103.0 [88.6; 119.5]	107.4 [99.4; 124.7]	102.5 [72.9; 125.4]	112.4 [100.7; 126.4]

*Note*: Values are the percentage of predicted (%pred) according to age, height and sex and are presented as the mean (SD) or median [interquartile range]. Group 1, asymptomatic COVID‐19; group 2, mild COVID‐19; group 3, moderate COVID‐19; group 4, severe COVID‐19; group 5, critical COVID‐19. Abbreviations: *D*_L,CO,5s_, pulmonary diffusing capacity for carbon monoxide (during a 5 s breath‐hold); *D*_L,NO_, pulmonary diffusing capacity for nitric oxide; *D*_M_, alveolar–capillary membrane diffusing capacity; STS, 30 s sit‐to‐stand test; *V*_C_, pulmonary capillary blood volume; 6MWT, 6 min walk test.

**TABLE 4:** Changes in pulmonary diffusing capacity and physical performance between 5.7 and 12.5 months follow‐up.

		All groups (*n* = 87)		Group 1 (*n* = 1)	Group 2 (*n* = 14)		Group 3 (*n* = 14)		Group 4 (*n* = 41)		Group 5 (*n* = 17)	
Parameter	*n*	Difference [95% CI]	*P*‐value	Difference	Difference [95% CI]	*P*‐value	Difference [95% CI]	*P*‐value	Difference [95% CI]	*P*‐value	Difference [95% CI]	*P‐*value
*D*_L,NO_%pred	87	3.4 [1.8; 4.9]	<0.001	2.5	5.1 [2.1; 8.3]	0.001	−0.8 [−4.3; 2.6]	0.63	3.1 [0.7; 5.6]	0.012	6.1 [3.1; 9.4]	<0.001
*D*_LCO,5s_%pred	87	2.9 [1.5; 4.3]	<0.001	−0.5	2.8 [−1.1; 6.2]	0.13	0.6 [−2.4; 3.5]	0.68	2.4 [0.4; 4.4]	0.017	6.2 [2.7; 9.3]	<0.001
*V*_C_%pred	41	2.3 [−0.4; 5.0]	0.087	2.2	0.8 [−14.9; 16.5]	0.90	0.4 [−5.4; 6.2]	0.88	3.3 [−0.6; 7.1]	0.093	3.0 [−2.3; 8.3]	0.21
*D*_M_%pred	41	1.9 [−1.7; 5.6]	0.29	2.6	1.1 [−7.6; 9.8]	0.76	−4.6 [−19.8; 10.7]	0.49	2.9 [−2.4; 8.1]	0.27	6.3 [−1.8; 14.4]	0.11
STS%pred	36	13.4 [6.8; 22.1]	<0.001	21.2	5.1 [−11.2; 12.7]	0.29	16.0 [2.9; 38.5]	0.064	15.2 [6.1; 32,7]	0.012	10.7 [−16.6; 27.6]	0.32
6MWT%pred	37	10.9 [6.8; 16.3]	<0.001	4.8	8.9 [−1.0; 18.4]	0.52	10.3 [2.6; 20.1]	0.018	10.8 [4.3; 21.6]	0.010	15.1 [1.5; 28.7]	0.025

*Note*: Changes are the percentage of predicted (%pred) according to age, height and sex and presented as mean [95% confidence interval (CI)]. Group 1, asymptomatic COVID‐19; group 2, mild COVID‐19; group 3, moderate COVID‐19; group 4, severe COVID‐19; group 5, critical COVID‐19. Abbreviations: *D*_L,CO,5s_, pulmonary diffusing capacity for carbon monoxide (during a 5 s breath‐hold); *D*_L,NO_, pulmonary diffusing capacity for nitric oxide; *D*_M_, alveolar–capillary membrane diffusing capacity; STS, 30 s sit‐to‐stand test; *V*_C_, pulmonary capillary blood volume; 6MWT, 6 min walk test.

### 3.3 Alveolar–capillary reserve

At ∼12.5 months after COVID‐19 infection, 11 patients [mean age 53.5 (13.9) years; four females; two in group 2, two in group 3 and seven in group 4], had an additional *D*_L,CO,NO_ measurement performed in the supine position. No alterations in *D*_L,NO_ or *D*_L,CO,5s_ were seen between the upright and supine position, with a change of 0.48 (−1.61; 2.56) mmol min^−1^ kPa^−1^ (*P* = 0.68) and 0.40 [−0.15; 0.95] mmol min^−1^ kPa^−1^ (*P* = 0.20), respectively. Likewise, no changes were observed in either *D*_M_ or *V*_C_, with a change of −0.25 (−2.76; 2.26) mmol min^−1^ kPa^−1^ (*P* = 0.85) and a change of 3.13 [−3.45; 9.71] mL (*P* = 0.40), respectively.

### 3.4 Physical performance

Of the 148 patients included, 131 (89%) completed functional tests at 5.7 months follow‐up, and 43 (29%) at 12.5 months follow‐up. At 5.7 months, STS and 6MWT were reduced similarly across the remaining severity groups (Table 3). In the 43 patients who completed 12.5 months follow‐up, STS had improved at 5.7 months, primarily driven by an improvement in group 4 (Table 4). For 6MWT, all groups had normal scores at both follow‐ups, but it was higher in groups 3–5 at 12.5 than at 5.7 months follow‐up (Table 4). At 12.5 months, 30 of those with an available *D*_L,CO,NO_ measurement had STS performed, and 31 had both a *D*_L,CO,NO_ measurement and 6MWT performed. No statistically significant correlations were observed between changes in *D*_L,CO,5s_%pred and STS (*r* = −0.10, *P* = 0.60), *D*_L,CO,5s_%pred and 6MWT (*r* = 0.14, *P* = 0.47), *D*_L,NO_%pred and STS (*r *= −0.12, *P* = 0.54) or between *D*_L,NO_%pred and 6MWT (*r* = 0.08, *P* = 0.69).

## 4 DISCUSSION

The main findings of the present prospective cohort study are that ∼60% of COVID‐19 patients exhibited at least one abnormal *D*_L,CO,NO_ metric at 5.7 months follow‐up. In the subgroup of patients with an abnormal *D*_L,CO,NO_ metric at 5.7 months follow‐up, both *D*_L,NO_%pred and *D*_L,CO,5s_%pred had increased at 12.5 months follow‐up, but still remained abnormal in two‐thirds of the patients. Concurrently, an improvement in physical performance according to both STS and 6MWT was observed, but with no apparent relationship to any *D*_L,CO,NO_ metric according to correlational analyses.

Both *D*_L,NO_%pred and *D*_L,CO,5s_%pred differed significantly at 5.7 months in an apparently severity‐dependent fashion. Hence, they were mostly reduced in groups 3–5 (Table 3) and accompanied by a classic restrictive lung function pattern with reduced TLC (Table 1). A comparable severity‐dependent reduction in *D*_L,CO,10s_ at 5.7 months follow‐up has likewise been linked directly to a restrictive lung disease pattern with a decreased TLC, ground glass opacities and fibrosis‐like changes in the lungs on high‐resolution chest CT imaging, indicating ongoing interstitial inflammation and remodelling (Nowers et al., 2002; Verleden et al., 2023). Although it is evident that a pathologically reduced *D*_L,CO,10s_ is exceedingly common post‐COVID‐19, the present findings indicate that *D*_L,NO_, *D*_L,CO,5s_, *D*_M_ and *V*_C_ are affected in a similar manner. This is in contrast to findings from two previous studies on 94 and 200 patients from Italy and Spain, respectively (Barisione & Brusasco, 2021; Núñez‐Fernández et al., 2021). In those two studies, *D*_L,CO,NO_ metrics were abnormal in 30%–60% of cases up to 9 months follow‐up, and reduction in *D*_M_ was found to be the dominant mechanism. However, in accordance with our findings, a study from Australia on 49 patients found that *D*_M_ and *V*_C_ were equally affected at 2 and 4 months follow‐up (Seccombe et al., 2023), indicating that neither pathological changes in the alveolar–capillary membrane nor in the blood available for gas exchange dominates as the underlying mechanism. These variances in study outcomes are not attributable to disparities in COVID‐19 severity or follow‐up durations, because these elements are comparable across the studies. Nonetheless, directly comparing the prevalence of abnormal *D*_L,CO,NO_ measurements between countries is challenging, owing to variations in the magnitude of the COVID‐19 outbreak, health‐care capabilities and a range of preventive, diagnostic and therapeutic approaches, including criteria for hospital and intensive care unit admissions. Despite these differences, it can be deduced that a significant reduction in *D*_L,NO_ and *D*_L,CO,5s_ at both 5.7 and 12.5 months post‐COVID‐19 is remarkably frequent, and its prevalence escalates in tandem with the severity of the acute phase of the disease. However, definitive conclusions about the specific alterations in *D*_M_ and *V*_C_ remain elusive at this time.

As for the gradual improvement in *D*_L,NO_%pred and *D*_L,CO,5s_%pred from 5.7 to 12.5 months follow‐up, this is in agreement with previous studies on *D*_L,CO,10s_, which generally show improvements over time in most patients with mild‐to‐moderate disease, while a concomitant remission of ground glass opacities and fibrosis‐like changes are observed on chest imaging (Huntley et al., 2022; Lee et al., 2022). However, it must be noted that the observed changes in the present study showed no apparent relationship to disease severity, and in any event, the absolute improvement was miniscule. Hence, *D*_L,NO_ showed an increase of 0.5–1.7 mmol min^−1^ kPa^−1^ and *D*_L,CO,5s_ of 0.1–0.3 mmol min^−1^ kPa^−1^ (see Online Supplemental File, Table C), both of which are well below the smallest real difference of 5.4 mmol min^−1^ kPa^−1^ for *D*_L,NO_ and 1.0 *D*_L,CO,5s_ as established in a recent test–retest reliability study on healthy volunteers (Madsen et al., 2023). Neither *D*_M_ or *V*_C_ showed any consistent changes from 5.7 to 12.5 months follow‐up, thus indicating that the changes in diffusing capacity with time were not dominated by either one in any of the severity groups.

Although *D*_L,CO,10s_ abnormalities have been linked mechanistically to persistent symptoms of dyspnoea and functional limitations as part of the long‐COVID syndrome (Schwendinger et al., 2022), we did not find any association between changes in *D*_L,NO_ or *D*_L,CO,5s_ with either STS or 6MWT. However, it must be noted that the main improvement was observed in STS, probably because most patients already had a normal 6MWT at 5.7 months. In any event, the main mechanisms of impaired exercise capacity after COVID‐19 typically do not reside in the lungs, but rather in the circulation (Baratto et al., 2021), and are likely to involve a combination of deconditioning and disease‐specific mechanisms (Jahn et al., 2022; Rinaldo et al., 2021). Accordingly, it has recently been documented that although targeted rehabilitation with high‐intensity interval training might reduce symptom severity and improve physical performance in many cases after COVID‐19, this is not associated with any changes in *D*_L,CO,10s_, but rather an increase in left ventricular mass (Rasmussen et al., 2023). In any event, relatively few patients were included in the regression analysis reported here, and there is therefore a high risk of type II error (Berg et al., 2024), thus precluding any firm conclusions regarding changes in *D*_L,NO_ or *D*_L,CO,5s_ and physical performance after COVID‐19.

The study has several limitations impacting its generalizability. Initially, although an invitation was extended to all patients discharged from University Hospital Copenhagen—Rigshospitalet, the analysis excluded certain patient demographics, notably those with dementia and residents of elderly care facilities. These groups are typically more susceptible to severe COVID‐19 and, potentially, more pronounced long‐term effects. In contrast, individuals experiencing symptoms of COVID‐19 might have a higher likelihood of participation. Additionally, a considerable number of patients opted out of the study. Notably, among patients who did not require hospitalization, there was a disproportionately high number of health‐care workers. Methodologically, there are also several issues to consider. Firstly, systematic errors are introduced to *D*_M_ and *V*_C_, because their measurement and derivation involves several assumptions and empirical constants (Borland & Hughes, 2020). For example, the prevailing scientific consensus acknowledges the diffusivity ratio, α, as 1.97, representing the ratio of physical solubilities of NO and CO in tissue (Wilhelm et al., 1977). Several studies have challenged this value, with some proposing higher α values to reconcile discrepancies between different measurement methods. However, these propositions are predominantly dismissed because they deviate from the physical diffusivity ratio, leading to inconsistent α values (Zavorsky et al., 2017). Furthermore, θ_NO_ is assumed to have a finite value, but was historically presumed infinite owing to its rapid reaction rate with free haemoglobin. However, comprehensive debates and recent studies have contested this assumption, establishing θ_NO_ as finite, with 1.51 mL blood min^−1^ kPa^−1^ (mmol NO)^−1^ providing the best current estimate, because it aligns well with theoretical predictions and with extensive in vitro and in vivo experimentation (Zavorsky et al., 2017). Likewise, the equations for θ_CO_ are based on empirical constants obtained at pH 7.4, rejecting other proposed values based on less accurate and non‐physiological pH measurements (Forster, 1987). However, for physiological interpretation, it must be kept in mind that for the calculation of θ_CO_, pulmonary capillary oxygen tension is assumed to be 13.33 kPa, although alveolar oxygen tension might cause it to fluctuate through between‐day and between‐manoeuvre variations in ambient pressure and alveolar ventilation. Nevertheless, none of the relationships embedded in these empirical constants and derivations is markedly affected by changes in oxygen tension. Until more substantial evidence is presented, it has been recommended that these empirical constants and derivations for α, θ_NO_ and θ_CO_ are retained (Zavorsky et al., 2017). Secondly, when it comes to assessing alveolar–capillary reserve by placing patients in the supine posture, this approach has recently been shown to exhibit high test–retest reliability. However, because the underlying changes in cardiac output are modest, it might provide a more representative measure of the different *D*_L,CO,NO_ metrics during submaximal exercise (Madsen et al., 2023), which was not attempted in the present study.

In conclusion, among the ∼60% of COVID‐19 patients who exhibited at least one abnormal *D*_L,CO,NO_ metric at ∼6 months follow‐up, there was no consistent pattern in terms of underlying contributions from *D*_M_ or *V*_C_. Furthermore, although *D*_L,NO_%pred and *D*_L,CO,5s_%pred did improve at ∼12.5 months, the changes were miniscule and neither physiologically nor clinically significant, and they appeared to be unrelated to any changes in physical performance. It remains indeterminate whether post‐COVID changes (or lack thereof) in *D*_L,CO,NO_ are mechanistically linked to changes in physical performance.

## AUTHOR CONTRIBUTIONS

Anna Agnes Lytzen: First draft, data interpretation, revisions. Thora Wesenberg Helt: First draft, data analysis, data interpretation, prepared tables, revisions. Jan Christensen: Data collection, data interpretation, revisions. Thomas Kromann Lund: Data collection, data interpretation, revisions. Anna Kalhauge: Data collection, data interpretation, revisions. Frederikke Rönsholt: Data collection, data interpretation, revisions. Daria Podlekavera: Data collection, data interpretation, revisions. Elisabeth Arndal: Data collection, data interpretation, revisions. Anne‐Mette Lebech: Conception, data collection, data interpretation, revisions. Birgitte Hanel: Conception, data collection, data interpretation, revisions. Terese L. Katzenstein: Conception, data collection, data interpretation, revisions. Ronan M. G. Berg: First draft, data analysis, data interpretation, revisions, supervision. Jann Mortensen: Conception, data collection, data interpretation, revisions, supervision.

## CONFLICT OF INTEREST

The authors declare that there are no conflicts of interest.

## Supporting information


**Online Supplemental Table A: Pulmonary diffusing capacity and physical performance at 5.7 and 12.5 months follow‐up**. Values are shown as absolute values and presented as the mean (SD) or median [interquartile range]. Group 1, asymptomatic COVID‐19; group 2, mild COVID‐19; group 2, moderate COVID‐19; group 4, severe COVID‐19; group 5, critical COVID‐19. Abbreviations: *D*_L,CO,5s_, pulmonary diffusing capacity for carbon monoxide (during a 5 s breath‐hold); *D*_L,NO_, pulmonary diffusing capacity for nitric oxide; *D*_M_, alveolar–capillary membrane diffusing capacity; STS, 30 s sit‐to‐stand test; *V*_C_, pulmonary capillary blood volume; 6MWT, 6 min walk test.
**Online Supplemental Table B: Pulmonary diffusing capacity and physical performance at 5.7 and 12.5 months follow‐up**. Values are shown as absolute *z*‐scores according to sex, age and height, and presented as the mean (SD) or median [interquartile range]. Group 1, asymptomatic COVID‐19; group 2, mild COVID‐19; group 3, moderate COVID‐19; group 4, severe COVID‐19; group 5, critical COVID‐19. Abbreviations: *D*_L,CO,5s_, pulmonary diffusing capacity for carbon monoxide (during a 5 s breath‐hold); *D*_L,NO_, pulmonary diffusing capacity for nitric oxide; *D*_M_, alveolar–capillary membrane diffusing capacity; STS, 30 s sit‐to‐stand test; *V*_C_, pulmonary capillary blood volume; 6MWT, 6 min walk test.
**Online Supplemental Table C: Changes in pulmonary diffusing capacity and physical performance between 5.7 and 12.5 months follow‐up**. Changes are absolute values, presented as the mean (95% confidence interval). Group 1, asymptomatic COVID‐19; group 2, mild COVID‐19; group 3, moderate COVID‐19; group 4, severe COVID‐19; group 5, critical COVID‐19. Abbreviations: *D*_L,CO,5s_, pulmonary diffusing capacity for carbon monoxide (during a 5 s breath‐hold); *D*_L,NO_, pulmonary diffusing capacity for nitric oxide; *D*_M_, alveolar–capillary membrane diffusing capacity; STS, 30 s sit‐to‐stand test; *V*_C_, pulmonary capillary blood volume; 6MWT, 6 min walk test.
**Online Supplemental Table D: Changes in pulmonary diffusing capacity and physical performance between 5.7 and 12.5 months follow‐up**. Changes are *z*‐scores according to sex, age and height, presented as the mean (95% confidence interval). Group 1, asymptomatic COVID‐19; group 2, mild COVID‐19; group 3, moderate COVID‐19; group 4, severe COVID‐19; group 5, critical COVID‐19. Abbreviations: *D*_L,CO,5s_, pulmonary diffusing capacity for carbon monoxide (during a 5 s breath‐hold); *D*_L,NO_, pulmonary diffusing capacity for nitric oxide; *D*_M_, alveolar–capillary membrane diffusing capacity; STS, 30 s sit‐to‐stand test; *V*_C_, pulmonary capillary blood volume; 6MWT, 6 min walk test.

## Data Availability

The data underlying our findings can be shared upon reasonable request directed to the corresponding author.
