# Supplementary material for: Pulmonary diffusing capacity for carbon monoxide and nitric oxide after COVID‐19: A prospective cohort study (the SECURe study)
Source: Exp Physiol. 2024 Mar 26;109(5):652–61. doi: 10.1113/EP091757 (PMC11061629; doi:10.1113/EP091757)
Supplement: Supplementary file 1 — Online Supplemental Table A: Pulmonary diffusing capacity and physical performance at 5.7 and 12.5 months follow‐up. Values are shown as absolute values and presented as the mean (SD) or median [interquartile range]. Group 1, asymptomatic COVID‐19; group 2, mild COVID‐19; group 2, moderate COVID‐19; group 4, severe COVID‐19; group 5, critical COVID‐19. Abbreviations: D L,CO,5s, pulmonary diffusing capacity for carbon monoxide (during a 5 s breath‐hold); D L,NO, pulmonary diffusing capacity for nitric oxide; D M, alveolar–capillary membrane diffusing capacity; STS, 30 s sit‐to‐stand test; V C, pulmonary capillary blood volume; 6MWT, 6 min walk test. Online Supplemental Table B: Pulmonary diffusing capacity and physical performance at 5.7 and 12.5 months follow‐up. Values are shown as absolute z‐scores according to sex, age and height, and presented as the mean (SD) or median [interquartile range]. Group 1, asymptomatic COVID‐19; group 2, mild COVID‐19; group 3, moderate COVID‐19; group 4, severe COVID‐19; group 5, critical COVID‐19. Abbreviations: D L,CO,5s, pulmonary diffusing capacity for carbon monoxide (during a 5 s breath‐hold); D L,NO, pulmonary diffusing capacity for nitric oxide; D M, alveolar–capillary membrane diffusing capacity; STS, 30 s sit‐to‐stand test; V C, pulmonary capillary blood volume; 6MWT, 6 min walk test. Online Supplemental Table C: Changes in pulmonary diffusing capacity and physical performance between 5.7 and 12.5 months follow‐up. Changes are absolute values, presented as the mean (95% confidence interval). Group 1, asymptomatic COVID‐19; group 2, mild COVID‐19; group 3, moderate COVID‐19; group 4, severe COVID‐19; group 5, critical COVID‐19. Abbreviations: D L,CO,5s, pulmonary diffusing capacity for carbon monoxide (during a 5 s breath‐hold); D L,NO, pulmonary diffusing capacity for nitric oxide; D M, alveolar–capillary membrane diffusing capacity; STS, 30 s sit‐to‐stand test; V C, pulmonary capillary blood volume; 6MWT, 6 min walk test. [file EPH-109-652-s001.docx]

**Online Supplemental Table A:Pulmonary diffusing capacity and physical performance at 5.7 and 12.5 mo.. follow-up.** Values are shown as absolute values, and presented as mean (SD) or median [interquartile range]. Group I: asymptomatic COVID-19; Group II: mild COVID-19; Group III: moderate COVID-19; Group IV: severe COVID-19; Group V: critical COVID-19. D_L,NO_: pulmonary diffusing capacity for nitric oxide; D_L,CO,5s_: pulmonary diffusing capacity for carbon monoxide (during a 5s breath-hold); D_M_: alveolar-capillary membrane diffusing capacity; V_C_: pulmonary capillary blood volume; STS: 30-second sit-to-stand test; 6MWT: 6-minute walk test.

|  |  | All | Group I | Group II | Group III | Group IV | Group V |
| --- | --- | --- | --- | --- | --- | --- | --- |
|  | *n* | Mean (SD) /median [IQR] |  | Mean (SD) /median [IQR] | Mean (SD) /median [IQR] | Mean (SD) /median [IQR] | Mean (SD) /median [IQR] |
| D_L,NO_  (mmol*min^-1^*kPa^-1^) |  |  |  |  |  |  |  |
| 5.7 months | 148 | 30.9 (9.4) | 28.8 | 33.7 (9.8) | 31.8 (9.4) | 30.1 (9.5) | 27.6 (7.2) |
| 12.5 months | 87 | 29.9 (8.5) | 29.5 | 30.6 (8.3) | 29.5 (9.7) | 29.9 (8.9) | 29.7 (7.3) |
| D_L,CO,5s_  (mmol*min^-1^*kPa^-1^) |  |  |  |  |  |  |  |
| 5.7 months | 148 | 7.0 (2.1) | 6.7 | 7.7 (2.2) | 7.1 (2.0) | 6.8 (2.1) | 6.1 (1.5) |
| 12.5 months | 87 | 6.7 (1.9) | 6.6 | 6.8 (1.6) | 6.7 (2.2) | 6.7 (2.0) | 6.5 (1.7) |
| D_M_  (mmol*min^-1^*kPa^-1^) |  |  |  |  |  |  |  |
| 5.7 months | 120 | 25.7 [20.9; 33.6] | 22.8 | 25.0 [22.0; 35.9] | 24.5 [20.7; 35.7] | 27.7 [20.8; 33.8] | 22.1 [16.9; 29.5] |
| 12.5 months | 48 | 24.9 [19.6; 31.9] | 23.4 | 22.9 [16.7; 24.5] | 24.4 [17.4; 29.1] | 29.2 [19.4; 34.2] | 28.7 [24.7; 33.1] |
| V_C_  (mL) |  |  |  |  |  |  |  |
| 5.7 months | 120 | 49.7 (13.9) | 53.2 | 57.1 (12.3) | 54.0 (14.0) | 47.0 (13.8) | 42.3 (10.9) |
| 12.5 months | 48 | 46.8 (11.7) | 54.3 | 50.9 (4.1) | 44.5 (9.1) | 45.7 (14.5) | 48.0 (9.9) |

**Online Supplemental Table B:Pulmonary diffusing capacity and physical performance at 5.7 and 12.5 mo.. follow-up.** Values are shown as absolute z-scores according to sex, age, and height, and presented as mean (SD) or median [interquartile range]. Group I: asymptomatic COVID-19; Group II: mild COVID-19; Group III: moderate COVID-19; Group IV: severe COVID-19; Group V: critical COVID-19. D_L,NO_: pulmonary diffusing capacity for nitric oxide; D_L,CO,5s_: pulmonary diffusing capacity for carbon monoxide (during a 5s breath-hold); D_M_: alveolar-capillary membrane diffusing capacity; V_C_: pulmonary capillary blood volume; STS: 30-second sit-to-stand test; 6MWT: 6-minute walk test.

|  |  | All | Group I | Group II | Group III | Group IV | Group V |
| --- | --- | --- | --- | --- | --- | --- | --- |
|  | *n* | Mean (SD) /median [IQR] |  | Mean (SD) /median [IQR] | Mean (SD) /median [IQR] | Mean (SD) /median [IQR] | Mean (SD) /median [IQR] |

| D_L,NO_ z-score |  | |  |  |  |  |  |  |
| --- | --- | --- | --- | --- | --- | --- | --- | --- |
| 5.7 months | 148 | | -2.0 [-2.7; -1.2] | -2.3 | -1.6 [-2.3; -0.9] | -1.6 [-2.3; -0.8] | -2.0 [-2.6; -1.3] | -2.6 [-3.2; -1.8] |
| 12.5 months | 87 | | -2.0 [-2.7; -1.4] | -2.0 | -1.8 [-2.7; -1.4] | -2.1 [-2.9; -1.5] | -1.8 [-2.3; -1.1] | -2.4 [-3.2; -1.8] |
| D_L,CO,5s_ z-score |  | |  |  |  |  |  |  |
| 5.7 months | 148 | | -2.0 [-2.7; -1.2] | -2.0 | -1.7 [-2.2; -0.9] | -1.7 [-2.5; -0.9] | -2.2 [-2.7; -1.3] | -2.6 [-3.6; -2.1] |
| 12.5 months | 87 | | -2.2 [-2.8; -1.6] | -2.0 | -1.9 [-2.5; -1.4] | -2.3 [-3.0; -1.8] | -2.1 [-2.4; -1.7] | -2.7 [-3.3; -2.3] |
| D_M_ z-score |  | |  |  |  |  |  |  |
| 5.7 months | 120 | | -1.3 (1.2) | -2.2 | -1.1 (1.2) | -1.1 (1.3) | -1.2 (1.3) | -1.8 (1.0) |
| 12.5 months | 48 | | -1.5 (1.2) | -2.0 | -1.9 (0.9) | -1.7 (1.3) | -1.4 (1.3) | -1.6 (0.9) |
| V_C_ z-score |  | |  |  |  |  |  |  |
| 5.7 months | 120 | | -1.9 (1.4) | -1.6 | -1.2 (1.2) | -1.5 (1.2) | -2.1 (1.4) | -2.7 (1.4) |
| 12.5 months | 48 | | -2.2 (1.1) | -1.4 | -1.7 (0.6) | -2.2 (0.6) | -2.3 (1.4) | -2.7 (1.1) |
|  | |  |  |  |  |  |  |  |

**Online Supplemental Table C: Changes in pulmonary diffusing capacity and physical performance between 5.7 and 12.5 mo. follow-up.** Changes are absolute values, presented as mean (95%CI). Group I: asymptomatic COVID-19; Group II: mild COVID-19; Group III: moderate COVID-19; Group IV: severe COVID-19; Group V: critical COVID-19. D_L,NO_: pulmonary diffusing capacity for nitric oxide; D_L,CO,5s_: pulmonary diffusing capacity for carbon monoxide (during a 5s breath-hold); D_M_: alveolar-capillary membrane diffusing capacity; V_C_: pulmonary capillary blood volume; STS: 30-second sit-to-stand test as 6MWT: 6-minute walk test.

|  | All (n=87) | Group I (n=1) | Group II (n=14) | Group III (n=14) | Group IV (n=41) | Group V (n=17) |
| --- | --- | --- | --- | --- | --- | --- |
|  | Diff [95% CI]  p-value |  | Diff [95% CI]  p-value | Diff [95% CI]  p-value | Diff [95% CI]  p-value | Diff [95% CI]  p-value |
| D_L,NO_  (mmol*min^-1^*kPa^-1^) | 1.1 [0.5; 1.7] | 0.7 | 1.9 [0.5; 3.3] | -0.4 [-2.0; 1.2] | 0.9 [-0.2; 1.9] | 2.3 [0.9; 3.7] |
|  | p=0.001 |  | p=0.012 | p=0.56 | p=0.094 | p=0.004 |
| D_L,CO,5s_  (mmol*min^-1^*kPa^-1^) | 0.2 [0.1; 0.3] | 0.1 | 0.2 [-0.2; 0.5] | 0.1 [-0.2; 0.4] | 0.1 [-0.04; 0.3] | 0.6 [0.3; 0.9] |
|  | p=0.001 |  | p=0.28 | p=0.69 | p=0.14 | p=0.001 |
| D_M_  (mmol*min^-1^*kPa^-1^) | 0.6 [-0.6; 1.9] | 0.6 | 0.3 [-3.0; 1.7] | -1.7 [-4.5; 3.3] | 1.0 [-0.6; 3.4] | 1.8 [-0.6; 3.8] |
|  | p=0.38 |  | p=0.78 | p=0.37 | p=0.32 | p=0.11 |
| V_C_  (mL) | 1.2 [-0.6; 2.9] | 1.1 | -0.3 [-10.5; 9.8] | -0.2 [-3.6; 3.2] | 1.7 [-0.8; 4.1] | 2.6 [-1.4; 6.1] |
|  | p=0.19 |  | p=0.94 | p=0.87 | p=0.17 | p=0.17 |

**Online Supplemental Table D: Changes in pulmonary diffusing capacity and physical performance between 5.7 and 12.5 mo. follow-up.** Changes are z-scores according to sex, age, and height, and presented as, presented as mean (95%CI). Group I: asymptomatic COVID-19; Group II: mild COVID-19; Group III: moderate COVID-19; Group IV: severe COVID-19; Group V: critical COVID-19. D_L,NO_: pulmonary diffusing capacity for nitric oxide; D_L,CO,5s_: pulmonary diffusing capacity for carbon monoxide (during a 5s breath-hold); D_M_: alveolar-capillary membrane diffusing capacity; V_C_: pulmonary capillary blood volume; STS: 30-second sit-to-stand test as 6MWT: 6-minute walk test.

|  | All (n=87) | Group I (n=1) | Group II (n=14) | Group III (n=14) | Group IV (n=41) | Group V (n=17) |
| --- | --- | --- | --- | --- | --- | --- |
|  | Diff [95% CI]  p-value |  | Diff [95% CI]  p-value | Diff [95% CI]  p-value | Diff [95% CI]  p-value | Diff [95% CI]  p-value |
| D_L,NO_ z-score | 0.3 [0.1; 0.4] | 0.3 | 0.5 [0.2; 0.8] | -0.1 [-0.4; 0.2] | 0.3 [0.05; 0.5] | 0.5 [0.3; 0.7] |
|  | p<0.001 |  | p=0.001 | p=0.60 | p=0.015 | p<0.001 |
| D_L,CO,5s_ z-score | 0.2 [0.1; 0.4] | -0.03 | 0.2 [-0.1; 0.5] | 0.1 [-0.2; 0.4] | 0.2 [0.03; 0.4] | 0.5 [0.2; 0.8] |
|  | p<0.001 |  | p=0.12 | p=0.51 | p=0.014 | p<0.001 |
| D_M_ z-score | 0.1 [-0.2; 0.3] | 0.2 | 0.03 [-0.7; 0.7] | -0.5 [-1.6; 0.5] | 0.2 [-0.2; 0.5] | 0.3 [-0.2; 0.8] |
|  | p=0.66 |  | p=0.90 | p=0.26 | p=0.32 | p=0.15 |
| V_C_ z-score | 0.2 [-0.01; 0.4] | -1.4 | 0.02 [-1.1; 1.2] | 0.04 [-0.4; 0.5] | 0.3 [-0.03; 0.5] | 0.3 [-0.1; 0.7] |
|  | p=0.062 |  | p=0.96 | p=0.81 | p=0.080 | p=0.13 |
